# Application and Research of Laser Manufacturing Technology in Nanomaterials

**DOI:** 10.3390/nano16201308

**Published:** 2026-10-16

**Authors:** Pei Zuo

**Affiliations:** School of Mechanical and Electrical Engineering, Wuhan Institute of Technology, Wuhan 430205, China; zuopei1990@wit.edu.cn

## 1. Introduction

Laser manufacturing has become one of the most broadly applicable approaches for fabricating micro- and nanostructures. It features non-contact energy transfer, high spatial selectivity, and broad material compatibility, enabling flexible processing [1,2]. In recent years, the field has advanced toward function-oriented nanomanufacturing, where the objective extends beyond geometric precision to encompass the regulation of optical response, wettability, interfacial chemistry, antibacterial behavior, and overall device performance [2,3,4,5,6]. Meanwhile, laser manufacturing has also evolved into one of the most versatile and precisely controllable platforms for the synthesis, processing, and patterning of materials at the nanoscale [1,7,8].

These function-oriented capabilities benefit from the development of processing methods. For instance, through liquid-phase laser ablation or irradiation, colloids and self-supporting nanostructures have been synthesized, enabling the preparation of functional nanostructures with controllable structure type, size, morphology, and stoichiometric ratio [7,9,10]. Maskless two- and three-dimensional (3D) nanofabrication has been achieved through femtosecond laser direct writing and two-photon polymerization, breaking the diffraction limit for feature sizes and enabling functional devices [11,12,13,14]. Pulsed laser deposition (PLD) has achieved the controllable, high-precision, and even atomic-level thickness growth of complex oxides, multi-layer structures, and functional nanomaterials [15,16,17].

While significant progress has been made in laser nanomanufacturing, several aspects still require further exploration. For example, this field requires a universal mapping of process-structure-performance to connect laser parameters, transient energy redistribution, and multi-scale material responses in metals, dielectric materials, polymers, hybrid systems, and multilayer films. In addition, although high-resolution patterning has been repeatedly demonstrated, the coupling between nanoscale precision, large-area throughput, and high reproducibility remains a central challenge for practical application.

Against this backdrop, the Special Issue “Application and Research of Laser Manufacturing Technology in Nanomaterials” collects four research articles and one review paper, covering laser-processed functional surfaces for medical devices, large-area fabrication of anti-reflective microholes, laser-assisted synthesis of submicron gold particles, dynamic mask projection-based thin-film ablation, and ultrafast selective ablation of multilayer thin films. These works involve nanoscale structures, mechanisms, or characterizations, demonstrate the broad research scope of laser manufacturing technology in nanomaterials, and offer insights into the great potential of laser manufacturing for advancing nanomaterials toward functional applications. The published contributions are described in the next paragraphs.

## 2. An Overview of Published Papers

The review by Ziyi Xu et al. (contribution 1) provides a comprehensive and critical review of recent advancements in laser processing technologies for the fabrication of functional surfaces in medical devices. The review highlights the growing importance of laser processing in the design of functional surfaces for medical devices. Rather than focusing only on fabrication itself, the authors emphasize how laser-enabled micro- and nanostructuring can be used to tailor key surface functions, including cell response, antibacterial activity, corrosion resistance, friction behavior, and anti-adhesion performance. By bringing together recent progress and current challenges in multifunctional surface design, this contribution demonstrates the strong potential of laser manufacturing to support the development of next-generation biomedical devices.

The article by Yulong Ding et al. (contribution 2) reports a femtosecond laser temporal and spatial modulation technique for fabricating large-area anti-reflective microholes on magnesium fluoride (MgF_2_) windows. Their work shows how temporal and spatial beam modulation can improve both structural quality and processing throughput, enabling highly consistent microhole arrays over practical areas. It achieved the fabrication of microhole arrays with sub-micron precision and surface roughness variations within 10 nm. The aspect ratio of the microholes was increased from 0.3 to 0.7 without compromising a processing speed of 10,000 holes per second. Large-area anti-reflective microholes were fabricated, forming a nanoscale refractive index gradient layer and achieving a transmittance increase to over 98%. This study illustrates how advanced ultrafast laser control can bridge the gap between high-precision micro/nanostructuring and scalable optical surface manufacturing.

The article by Ilya V. Baimler et al. (contribution 3) demonstrates a three-step method for the synthesis and production of submicron spherical gold particles using laser ablation in liquid (LAL), laser-induced fragmentation in liquid (LFL), laser-induced nanochain formation, and laser melting in liquid (LML). LAL and LFL produced 20 nm spherical gold nanoparticles. Subsequent 532 nm laser irradiation formed gold nanochains, and subsequent 1064 nm irradiation generated 50–200 nm spherical gold particles. Submicron gold particles formed rapidly and fully developed after 480,000 laser pulses. Larger particles were obtained at higher pulse energy, with fragmentation occurring above the threshold fluence. Submicron particle size increased linearly with initial colloid concentration up to a certain concentration. The study demonstrates that gold particle size evolution can be flexibly tuned by laser irradiation conditions and colloid concentration, offering a chemically clean approach to prepare stable gold particles over a broad size range. It highlights the versatility of laser-based synthesis for controllable nanomaterial production.

The article by Jonas Amann et al. (contribution 4) presents a scanning-based dynamic mask projection concept, combining both strategies by integrating a digital micromirror device (DMD) for dynamic binary amplitude mask generation with galvanometric scanning for high-speed lateral repositioning of the projected pattern. This concept was verified by single-pulse selective ablation of 10 nm TaN thin films on glass using 515 nm, 230 fs laser pulses. The optical system enabled a 770 nm minimum feature size across a scan field with an area-equivalent circular diameter of 550 µm. Dynamic mask projection combined with fast scanning offers a scalable route to high-throughput laser nanoprocessing. The work highlights the potential of dynamic optical control strategies for scalable laser patterning and micro/nanofabrication technologies, which can support application domains including metasurface manufacturing, high-throughput selective thin film processing, and long-term optical data storage.

The article by Sergey I. Ashitkov et al. (contribution 5) is focused on the ultrafast dynamics of layer-by-layer ablation of a multilayer Ni/Al thin film at the initial stage (0–120 ps) after irradiation with a single femtosecond laser pulse. By combining time-resolved interferometric observation with TTM-MD simulation, insight into the early-stage response of layered films was provided, including spallation, phase explosion, and transient material ejection. Partial removal of the 46 nm top Ni layer occurred via spallation at an expansion velocity of hundreds of meters per second. Its low spallation threshold arose from rarefaction-wave interference reflected at layer interfaces. Full removal of the Ni layer proceeded through phase explosion with jet-like material ejection, coupled with explosive expansion of the superheated molten Al underneath. This study contributes to a deeper mechanistic understanding of laser interactions with multilayer thin films and offers useful guidance for selective thin-film laser processing.

## 3. Conclusions

This Special Issue highlights the broad and rapidly expanding role of laser manufacturing technology in nanomaterials research. The collected papers demonstrate that laser-based approaches are no longer limited to precise material removal or pattern transfer, but are increasingly being used to enable multifunctional surfaces, high-efficiency micro/nanostructuring, controllable nanostructure synthesis, and mechanistic studies of ultrafast material responses. Together, these contributions reflect the versatility of laser manufacturing across different material systems and application scenarios, ranging from biomedical interfaces to optical components and thin-film processing.

In addition, the published works also point to several key directions for future research. First, further efforts are needed to establish clearer process-structure-performance relationships, especially for complex nanomaterials, multilayer films, and hybrid systems. Second, the combination of nanoscale precision with high throughput, large-area fabrication, and high reproducibility remains a significant challenge for practical applications. Third, more attention should be given to integrating laser processing with application-driven design, such as multifunctional biomedical surfaces, advanced photonic structures, and scalable nanomaterial synthesis. In this context, laser manufacturing is expected to evolve from a fabrication tool into a more comprehensive platform for designing, tailoring, and functionalizing nanomaterials. We hope that this Special Issue will stimulate further interdisciplinary research and accelerate the development of laser manufacturing technologies in nanomaterials toward broader functional and practical applications.
